# Updating the Breeding Philosophy of Wheat to Fusarium Head Blight (FHB): Resistance Components, QTL Identification, and Phenotyping—A Review

**DOI:** 10.3390/plants9121702

**Published:** 2020-12-03

**Authors:** Akos Mesterhazy

**Affiliations:** Cereal Research Non-Profit Ltd., 6726 Szeged, Hungary; akos.mesterhazy@gabonakutato.hu

**Keywords:** toxin overproduction, disease index, resistance to DON, resistance to FDK, Fusarium damaged kernel, deoxynivalenol, differing QTL functions, upgrading phenotyping of QTLs, resistance types, breeding for complex FHB resistance

## Abstract

*Fusarium* head blight has posed continuous risks to wheat production worldwide due to its effects on yield, and the fungus provides additional risks with production of toxins. Plant resistance is thought to be the most powerful method. The host plant resistance is complex, Types I–V were reported. From the time of spraying inoculation (Type I), all resistance types can be identified and used to determine the total resistance. Type II resistance (at point inoculation) describes the spread of head blight from the ovary to the other parts of the head. Therefore, it cannot solve the resistance problem alone. Type II QTL (quantitative trait locus) *Fhb1* on 3BS from Sumai 3 descendant CM82036 secures about the same resistance level as Type I QTL does on *5AS* and *5ASc* in terms of visual symptoms, FDK (Fusarium damaged kernel), and deoxynivalenol response. Recently, increasing evidence supports the association of deoxynivalenol (DON) content and low kernel infection with FHB (Fusarium head blight) resistance (Types III and IV), as QTL for individual resistance types has been identified. In plant breeding practice, the role of visual selection remains vital, but the higher correlations for FDK/DON make it possible to select low-DON genotypes via FDK value. For phenotyping, the use of more independent inocula (isolates or mixtures) makes resistance evaluation more reliable. The large heterogeneity of the mapping populations is a serious source of underestimating genetic effects. Therefore, the increasing of homogeneity is a necessity. As no wheat varieties exist with full resistance to FHB, crops must be supported by proper agronomy and fungicide use.

## 1. Introduction

Fusarium head blight (FHB) is one of the main diseases of wheat, and the toxins produced by the fungus make it a feared problem. FHB is a very complex disease of wheat; many genetic and non genetic effectors modify disease response [1,2,3,4]. In the review, we concentrate on three important points that were not in the centrum of the research, therefore they were rather neglected in the past decades (problems in resistance evaluation, the resistance types, and the phenotyping problems in the quantitative trait locus (QTL) analyses and recognition of their role in forming genetic responses). However, they are important for breeding and *Fusarium* research in wheat and other small grains. This treatise is not intended to evaluate the molecular biology methodologies as QTL (quantitative trait locus) analysis, genome wide association mapping, and other methods. All depend on the quality and reliability of phenotyping. As we have a long experience with working with four or more isolates in a test [5,6], and in all tests, we evaluated visual symptoms as disease index (DI), FDK (Fusarium damaged kernel), and DON (deoxynivalenol), we come to conclusions that can be useful also in other research groups. 

In the phenotyping (resistance studies, genetic analyses, host-pathogen relation studies, and all tests that need artificial inoculation), normally one inoculum is used. This can be a conidium suspension from a single isolate, can be a mixture of different isolates, can be mycelium or a mixture of conidia and mycelium, and also ascospores can be used. Many authors use mixtures of different isolates [5] or a single isolate [6]. The authors cited in [5,6] used very variable conidium concentrations, without any reference to aggressiveness. The resistance tests were conducted with single isolates or mixtures by spraying or point inoculation, and in several experiments, both were applied [7,8,9,10,11,12,13,14,15,16,17,18,19,20].

Gadaleta et al. [21] used spraying inoculation and visual symptoms were recorded. In the papers, incidence (% of the infected spikes), severity (the extent of infection within spike), and disease index (DI) were used. They evaluated the extent of the infection of the spike, e.g., severity, and this was addressed as Type II resistance. However, Type II resistance can be identified only by point inoculation. Spreading of the infection is a general phenomenon in diseases, and during epidemics, steadily growing numbers are recorded. Type I resistance has this spreading function, which causes issues. Ma et al. [22] underlines this problem and stresses the problem of identification of Type I resistance, which was also mentioned by Dill-Macky [23]. The lack of testing for FDK and DON is another problem. Lemmens et al. [24] say that the final goal of breeding is to decrease the amount of fungal toxins. However, they added that mycotoxin analysis is normally not conducted as it is costly, the same for FDK analysis, and only at the end of the breeding process will a toxin test be performed, if at all. Our problem is that QTL identifications are done for genetic work, and except for several QTL, we never can be sure that a visual symptom reduction is followed by toxin reduction. As QTLs were identified with different effects on visual symptoms, FDK, and DON, there is a chance to observe fewer symptoms but no reduction in DON [25] or even an increase in DON [26] (toxin overproduction). Schroeder and Christensen [27] described Type II resistance (resistance to spreading) as a response to the point or single floret inoculation. However, the spreading following spray inoculation cannot be described as Type II resistance as done in [21]. The spreading function is also found in Type I resistance (area under disease progress curve) [28]. For this and other reasons, we have to rethink the problem of the resistance types/components.

Liu et al. [29] used corn grains infected by a mixture of 20 *F. graminearum* strains with chemotypes 3- and 15-ADON (acetyl DON) for spawn inoculation. As heading date was variable (no information on the extent), the early genotypes had much longer irrigation times than the later ones, which can cause a stronger infection in the earlier genotypes. Such an analysis has not been conducted. FDK and DON were not tested. This is a problem as farmers want to have low DON. 

Mendes et al. [30] tested FDK and DON against *F*. *graminearum* and *F. meridionale*, which is a nivalenol (NIV) producer. It is important that severity (meaning here disease index) against *F. graminearum* was between 99.2% and 100% without significant difference, and the less pathogenic *F. meridionale* had between 64.6% and 97.1% severity, but the FDK was very diverse with values between 21% and 97%. DON values were between 45 and 691 mg/kg, and NIV values were between 4.3–355 mg/kg. The data are not supported by LSD values or LSD values were not calculated. Their data show clearly that the three traits are not synonyms. In cultivar Quartzo, 691 mg/kg DON was measured at 99.8% severity with a very strong DON overproduction. In CD105, 45 mg/kg DON was found, but NIV had 355 mg/kg, which seems to have been also an overproduction. The results of [30] seem to support the toxin overproduction proved earlier [26]. This aspect was not discussed in the paper [30]. It is also important that the FHB resistance cannot be characterized only by the visual symptoms. The data show large variations, such as that in FDK, the difference between 97.1% and 40.6% was not statistically significant. This shows that a much higher amount of data is required, but we can suppose methodical problems were also a factor. 

Amarashinghe [31] is another exception; she had seven isolates in a fungicide test with highly susceptible and moderately resistant wheat varieties. The results of the 3-ADON and 15-ADON isolates were pooled, and an analysis to measure possible differences between isolates has not been conducted. The cultivar/isolate interaction was significant in 2009, identifying different responses of the cultivars to the different isolates, but in 2010, no significant interaction was found. It is important that both FDK and DON were analyzed. Of course, the possibility was there to view the individual isolates separately, but this analysis was not conducted. As the publication did not contain such data, further analysis was impossible. Chen et al. [32] worked with three isolates separately, including three replicates and two plants, by point inoculation. Only visual symptoms were recorded and were pooled, so the data according to isolates could not be analyzed.

Many QTL and resistance analyses were conducted using a single isolate at very different conidium concentrations [8,14,16,17,19,20,33], or by mixtures [5]. 

For these reasons, the objectives of the paper are:

1. Summarize the experimental methods and their uses for phenotyping supplemented with FDK and DON measurements and symptom evaluation.
2. Evaluation of new developments in determining Type I and Type II resistance and their significance for breeding and research.
3. The role of Type III (FDK) and Type IV (DON) in the resistance of FHB in wheat.
4. The role of QTL in the resistance to different traits (DI, FDK, DON) and influence of flowering variation on the mapping QTLs and its influence on the QTL performance.
5. How the breeding of more resistant cultivars can be enhanced by the new insights.

## 2. Advances in Phenotyping

As shown before [5,6], the general praxis is that the artificial inoculations are made nearly exclusively with one inoculum from a single isolate or a mixture of different isolates. Therefore, the first issue is determining if the response against one inoculum can properly describe the amount of resistance and the differences in resistance. In about 70% of the studies, only visual rating was done. The question is, how well can the visual rating describe FHB resistance? The data show that the very variable conidium concentrations applied led to very different disease severities. However, papers applying the same conidium concentration cannot secure a similar level of infection. So, an arbitrary chosen conidium concentration could not regulate the aggressiveness level in the tests, but the amount of inoculum inoculated indeed affect severity of the disease. This is not surprising as there are genetic differences in aggressiveness between isolates, but the resistance level is also different. As no aggressiveness control was made, the regulation of the conidium concentration had no experimental basis. Experimental data prove that the dilution influences the aggressiveness of the inoculum differently for different isolates and its influence cannot be predicted [6]. The mixing of isolates is a similar situation. The result of the mixing cannot be predicted well, and the aggressiveness of the mixture never reached the aggressiveness of the most aggressive component [5], but could balance to some extent the low-aggressiveness partner in the mixture. 

**Figure 1:**
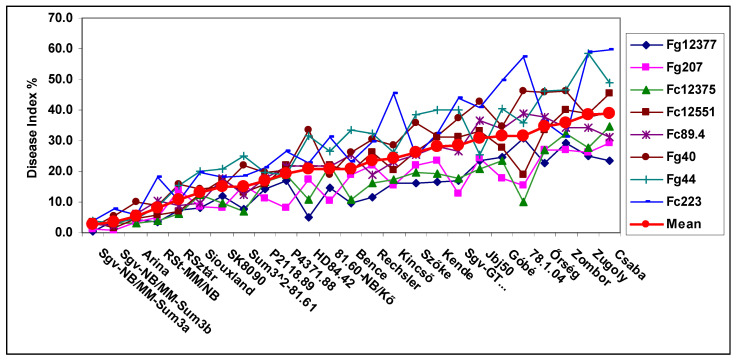
Resistance of winter wheat lines to *F. graminearum* and *F. culmorum* isolates 1994–1996. Disease index, [34]. Larger variations in surveying of the susceptible cultivars could suggest that the disease development is more variable in susceptible cultivars due to a mixture of factors including the genotypes. The most resistant ones have high stability and low variation.

Mesterhazy et al. [34] conducted tests with eight isolates of *F. graminearum* and *F. culmorum*. The results were not evaluated in this respect, so we concentrated on the means. The analysis has further lessons. Figure 1 shows the disease index data for the eight isolates. Among the genotypes, several represented highly resistant ones from Sumai 3 (Sum3) and Nobeoka Bozu (NB) backgrounds (FHB breeding program, Szeged). The more resistant genotypes showed lower variation below five, but 78.1.04 had a variance of 262. As in the published data, only one inoculum was used, and the figure clearly shows that the genotypic differences had a large variation for the different isolates. There is only a low reliability of the identification of medium or lower effective QTLs. The large genotype differences can be identified with a large probability when aggressiveness is high. There is an exception; GK Zombor had a variance of only 56 compared to 262 of line 78.0.04. This can explain the artifacts in the QTL analyses and the false resistance or susceptibility classification of the given genotype. The means of isolates shows the level of resistance better than any of the single isolates. The number of correlations between cultivar reactions to the eight isolates was significant in 112 cases of the three hundred, 37%. This shows well the differing responses of the genotypes. 

**Figure 2:**
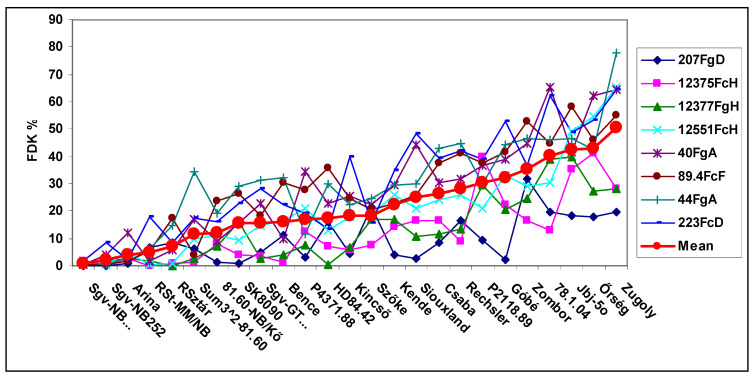
Resistance of winter wheat lines *F. graminearum* and *F. culmorum* isolates 1994–1996. Fusarium damaged kernels (FDK), percentage, [34]. Larger variations in surveying of the susceptible cultivars could suggest that the disease development is more variable in susceptible cultivars due to a mixture of factors including the genotypes. The most resistant ones have high stability and low variation.

The FDK data (Figure 2) show a similar picture with the difference that even among susceptible cultivars there can be reactions close to zero. The variation in response to different isolates is larger than that seen in the disease index. The variation is also higher. The two most resistant lines had their variance below 10 (0.4 and 8.9), and the highest was found in GK Zugoly, 472. The mean of the eight isolates give a much more reliable resistance ranking than any of the isolates separately (n = 8). In FDK, 85 correlations were significant from the 300, 28%. 

**Figure 3:**
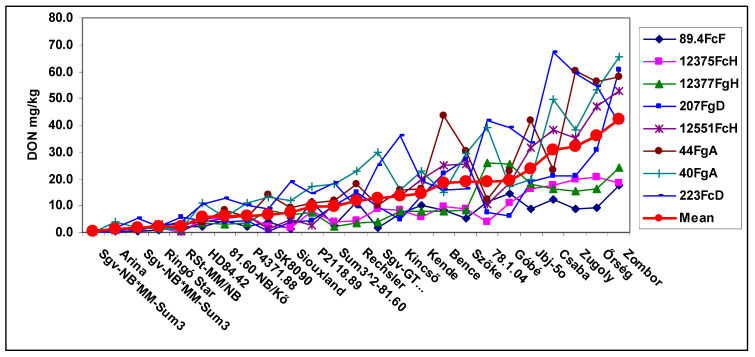
Resistance of winter wheat lines and cultivars to *F. graminearum* and *F. culmorum* isolates, 1994–1996. Deoxynivalenol (DON) contamination, mg/kg [34]. Larger variations in surveying of the susceptible cultivars could suggest that the disease development is more variable in susceptible cultivars due to a mixture of factors including the genotypes. The most resistant ones have high stability and low variation.

The DON response of the cultivars to the eight isolates (Figure 3) presents a similar picture, as shown in Figure 1 and Figure 2. As susceptibility grows, the variation showed an increasingly wider stripe. Five genotypes had lower variance than five did, two have value between 5 and 10, and the highest number was 393. The higher the resistance is, the lower the variance. Seventy-nine correlations (26%) of the possible 300 showed significant correlation at *p* = 0.05 or higher. This means that the high resistance gives a much better chance to achieve lower or low DON contamination in all epidemic situations. For the most susceptible genotypes, any value can be found between 5 and 40 or 9 and 59 mg/kg. This confirms earlier data [3,34].

The correlations between traits for each isolate were counted for the 25 genotypes (Table 1). The DI/FDK correlations varied between r = 0, 64 and r = 0.86, the DI/DON correlation ranked from r = 0.74 to r = 0.87, and the FDK/DON correlations varied between r = 0.69 and r = 0.89. The patterns seem to be different, and when considering the correlations between experimental means, DI/FDK gives r = 0.84, the DI/FDK presents r = 0.89, and FDK/DON shows r = 0.81. Similar patterns were found [35], but significantly higher correlations were found for FDK/DON than for DI/DON. In other tests, different results were found; the DI/DON had much lower values than the FDK/DON correlations [6,26,36,37,38]. 

The three traits shared several common features. The most resistant genotypes had low variation, and the medium and highly susceptible genotypes showed increasing deviations in responses to the different isolates. The genotype differences between isolates showed higher differentiation in the most aggressive isolates and in the lower-aggressive isolates, the differentiation was much lower and the genotype differences were less expressed. The genotype ranking between genotype reactions to the eight isolates was different, and the highest rate of significant correlations of the 300 possible correlations between the genotype responses to the eight isolates was found for DI (37%), while for FDK and DON, it was lower (26 and 28%). This means that the resistance ranking of the genotypes to the eight isolates showed a high variability for the isolates and so the resistance ranking to the individual isolates was not reliable enough to use it as a reliable phenotyping result. When there was only one inoculum, it could be any of the eight types. When they were analyzed in separate QTL analyses, we could see the differences. We will come back to this problem later. It is also clear that the mean of the eight isolates reflects the amount of the resistance more precisely than any of the isolates separately. This may cause a significant error in the phenotyping and can influence the QTLs to be identified. 

Based on the results, the level of the resistance should also be evaluated. When the genotypes are ranked at a low or medium aggressiveness level, even the most infected DI at 20% (Figure 1) can be considered to be considerably resistant on a scale of 0–100% DI or FDK, or highly susceptible, depending which isolate was used. 

**Figure 4:**
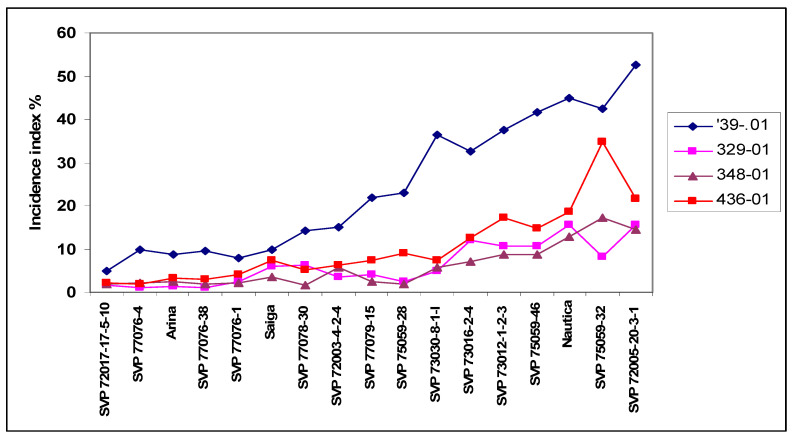
Incidence of 17 winter wheat genotypes against four isolates of *F. culmorum* across three years, IPO Wageningen, Snijders and van Eeuwijk [39].

Snijders and van Eeuwijk [39] made a similar test with 17 winter wheat genotypes and four independent isolates of *F. culmorum*. They presented only visual data as incidence, e.g., the percentage of diseased heads (Figure 4). The lessons are the same as presented in Figure 1. 

**Figure 5:**
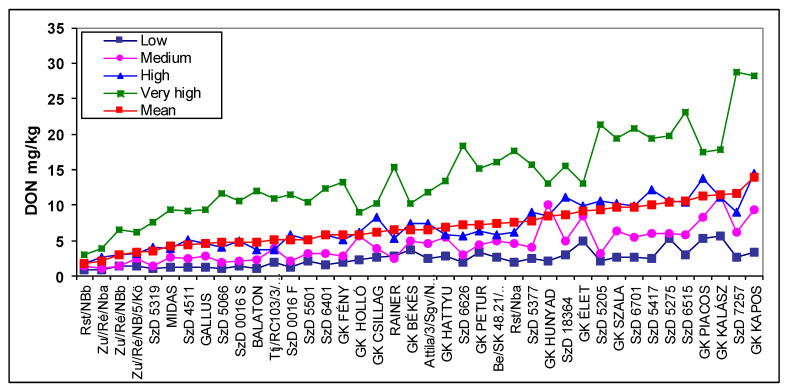
Deoxynivalenol (DON) data (mg/kg) in the methodical test for inoculation methods across years and methods according to aggressiveness level, 2009–2012 [26] LSD 5% for genotypes: 2.14, between any points in the chart (except genotype means) 4.28.

The reactions of the genotypes can also be grouped according to the visual records of the different isolates [26]. As the ranking of the isolates in aggressiveness is normally different between years, the influence of the aggressiveness level can be clearly evaluated. In this case, the lowest DI from the different isolates came from the low-aggressiveness group which includes 2–3 different isolates. The medium group contained the second largest data, and the same was applied to the high and very high-aggressiveness groups. The DON response of the genotypes is plotted in Figure 5. The DI and FDK data are shown in Figures S1 and S2. The highest aggressiveness showed the greatest expressed genotype differences in each case, but with different distances between aggressiveness levels. Lower genotype differences were seen with the high level of aggressiveness. The scientific value of the two lower aggressiveness levels was more moderate and the low level was close to useless. The common feature between Figure 3 and Figure 5 is that the mean data show the most useful data series and the amount of resistance seems to be the closest to reality. Several genotypes were identified at low aggressiveness that had unexpectedly high infection or DON rates. These data are useful when estimating risks as low epidemic levels occur more frequently. 

For inoculation methods, we use the spraying inoculation combined with 48 h polyethylene bag coverage following spraying in Table 9 in [26], which gave the best differentiation of the genotypes and verified the inoculation praxis done until now. Spraying inoculation combined by mist irrigation was less effective (reduction of DI of about 50%, reduction in FDK of 63%, and reduction in DON of 70%). This indicates the practical usefulness of the spraying and polyethylene (PE) coverage method, compared to the spraying method with misting, and the spawn method. The traits reacted differently; in DI, the three methods gave very close results. However, the FDK severity decreased by 74% using the spawn method, and the DON showed 85% reduction compared to the Spray + PE method, i.e., the least effective differentiation of the genotypes was performed here. 

Most breeding programs use spraying inoculation combined with some type of mist irrigation for mass selection; therefore, the usefulness of this screening should be tested, even if for scientific purposes the point inoculation method is used most often. This aspect will be discussed in the next chapter on resistance types. Of the traits tested, the DON was the most useful, and in most cases, this correlated better with FDK than with the visual symptoms. In this and other tests, we used four or eight isolates without mixing [35,40,41] and an isolate/year interaction was demonstrated in each experiment; therefore, we cannot speak about stability in aggressiveness. This agrees with the need of the growers and breeders to receive information about the risk of DON contamination. The differentiation of the genotypes is much better at high aggressiveness levels, therefore the use of more, in our case four or eight isolates, in research can balance the individually differing isolate data and can provide a higher accuracy (Figure 4) [26]. As a mean product of several isolates, the amount of the resistance and the ranking of genotypes can be demonstrated at higher reliability.

The conclusions are that (1) an aggressiveness test is useful to select the best inocula for infection. The research supported the view that previous experimental data are useful in the selection of the best inocula for experimentation. (2) The previous aggressiveness test of isolates for use proved to be a good practice [5,6,23,39,42,43]. (3) As tests of aggressiveness at the seedling stage show a significant correlation with FHB symptoms [5,6], tests to select more aggressive isolates for field usage can be performed. The same is true to screen inocula for field inoculations. It is important to identify the resistance differences for the identification of resistance level in parental lines or cultivars and for variety registration. For this work, four isolates were needed. The comparison of resistance within ripening groups was not a problem, because in each group, control cultivars or lines helped in the classification in field experiments. (4) For phenotyping in QTL, this method was suitable, but additional problems coming from the mostly wide flowering period and other problems should be resolved in future research; see the QTL chapter. (5) For mass selection, one aggressive isolate or mixture following an aggressiveness test can secure the disease pressure to perform an effective negative selection. 

## 3. Type I and Type II Resistance to Infection and Spreading

It is known that Type II resistance is only a part of the total resistance. However, Type I is normally neglected as people do not see clearly what is it and how it should be worked with. This can lead to susceptibility. The parents of Sumai 3 (Funo from Italy and Taiwanxiaomai from China) were moderate resistant genotypes [44], therefore *Fhb1* alone cannot secure high resistance) as supposed in many early papers having 3BS (*fhb1*) QTL [9]. Chrpova et al. [11] reported no significant difference between 3BS and no QTL plants (this could be genotype dependent), but the 3BS+5AS plants showed much better resistance. 

The famous paper written by Schroeder and Christensen [27] reported two resistance types, where Type I was the component for initial infection and response to spray inoculation, and Type II was the component designed for spreading following point inoculation. In the text, the terms resistance to infection and resistance to initial infection were used as synonyms. However, the spray and point inoculation were evaluated by using the same method, and according to the data, no difference in visual symptoms was found between Type I and Type II. The only difference was that the Type II resistance had much lower ratio in *Fusarium* damaged kernels (FDK). In both components, we can determine the time from inoculation to the appearance of first visual infection measured by the number of infected spikelets. For this, the inoculation time must be known exactly. This was the reason Schroeder and Christensen [27] applied artificial inoculation methods for both types; otherwise, the determination of time between inoculation and the appearance of the first (initial) symptoms could not be resolved. This meant that they [27] did not differentiate between incidence and severity; their numbers refer to disease index. The timing of the exact inoculation date is a problem of the natural epidemics, and the spawn method has a similar difficulty, as discussed by Dill-Macky [23]. Therefore, differentiation in this case was hardly possible. The role of FDK was later forgotten despite the emphasis on this issue. Ma et al. [45] characterize the situation “The inherent relationships or differences of distinct resistance types are far from clear”. I agree. Therefore, we need to develop more useful hypotheses to understand what is happening in the resistant and susceptible plants. 

In view of the evaluated text [27], we see that the first visually infected spikelet was observed and this was the initial infection. This information was obtained from the given data. This means that all other explanations can be considered as speculation. One could hypothesize that a physiological trait inhibits the first infection following germination of the *Fusarium* spores. Theoretically, this could be possible, but in the text, nothing was said to insinuate this solution. However, the same symptom was also observed for the point inoculation where a droplet of suspension was placed over the ovary in the wheat spikelet (in the middle of the head) at the lower floret [46]. In both resistance types, several evaluations were made and area under the disease progress curve (AUDPC) was counted. Later, only one reading was made on the 21st day after inoculation [47]. Working with a point inoculation (single floret inoculation), excellent correlation was found between AUDPC and this evaluation. Therefore, clarification should be made on the definition of Type I and II resistances, e.g., the difference between the effect of spraying and point inoculation. Researchers were not aware of Type I resistance [22,23], therefore, Type II was prioritized as it was easier to work with. Type II had clear definition and could be analyzed under controlled conditions that were hardly possible in field inoculation where large materials had to be handled. 

However, as Dill-Macky [23] (2003) remarked, Type II resistance and other types of resistances are hard to identify in field nurseries. This is mainly due to the fact that the secondary infections cannot be separated from the primary infection. Therefore, the inoculation date cannot be omitted. It should be added also that even without multiple infections, the problem is that the time of the first inoculation is also unknown. Without this, no AUDPC evaluation is possible. So, without artificial inoculation, the case cannot be solved. To this contributed the fact that Chinese research [46] started to apply point inoculation exclusively, and it seemed to be the right solution for the breeding problems [23]. We have to consider that Frontana was classified as Type II resistance [27], while in later research, it was characterized as Type I resistance [28] on chromosomes 3A, 1B, 2A, 2B, 4B, 5A, and 6B, but only a small effect of Type II QTL was also found. They characterized all others as resistance to penetration (Type I). In effect, this was resistance to initial infection in senso, Schroeder and Christensen [27]. Mardi et al. [48] came to the same conclusion identifying QTL in 1BL, 3AL, and 7AS explaining 7.9%, 7.7%, and 7.6% of the variation by spraying inoculation, so the original classification of Frontana could not be confirmed. It is also true that Schroeder and Christensen [27] tested only seven genotypes and one inoculum was used, and this is not a significant number to find consequent behavior schemes [5,6]. From their Table 1 [27], we can see that three inoculation dates were applied; interestingly, the latest inoculation date at soft dough stage gave the highest numbers in five of the seven genotypes, even normally the inoculation at flowering produces the highest infection [36]. There is no clear explanation for this in the text. The aggressiveness was not measured [27], but high conidium production was searched for in isolates. A range of 3–7 × 10^6^ conidia was used from *F. graminearum*; this is much higher than used by the present praxis. Therefore, a number of questions remained unanswered.

Cassini [49] mentioned the ongoing breeding program in France and Canada, but no information was given for resistance types. Cook [50] concluded that as the Fusaria infect many other plants than *Gramineae*, "there is little prospect for good or even moderate genetic resistance to these pathogens in wheat, barley or oats". Cook [51] reported briefly about the Chinese problems, but no useful information was given on resistance. However, Sumai 3 was registered at the beginning of the 1970s [44], so the negative opinions were not supported. 

It is also important to know the requirements. Some authors preferred incidence (percent of the heads showing visual symptoms) in mapping QTLs, some others severity (the rate of the spikelets infected in one ear is expressed in the number of spikelets or percentage), but only for the heads that showed infection [9]. In several cases, both were evaluated and the disease index, a multiplication of incidence, and severity were used. In older literature, severity often meant disease index [52], so the meaning of the terms also changed. However, the text shows in most cases the meaning of the term used. The trait that corresponds to the description of Schroeder and Christensen [27] is the disease index.

The first review article for wheat FHB QTLs [9] summarized the findings of 52 papers from the first decade. In their Table 1, 59 QTLs were identified for spreading (Type II), 59 for severity, and 13 for incidence (Type I). No data were shown for disease index, but in several populations, both severity and incidence were mapped [28]. In five cases, DON was mentioned and kernel resistance was mapped in six cases. In several cases, an overlap was shown, as the same QTL was identified by more authors. The number of validated QTLs is much smaller (independently proven in more mapping populations), 3BS, 6B, and 5A QTL have multiple proof by different authors [9]. In 3BS, there is multiple data on kernel infection, spread, and DON. In 5A, severity and DON were mapped together. As in the paper [9], the percentage of the explained variation is given, even the data from the same source of resistance can be different, indicating unknown background effects. Additionally, near isogenic or DH lines can diminish background effect. It is worth discussion, whether this is advantageous or disadvantageous. In this review, no papers were found to map spreading or severity with DON and kernel infection together. 

**Figure 6:**
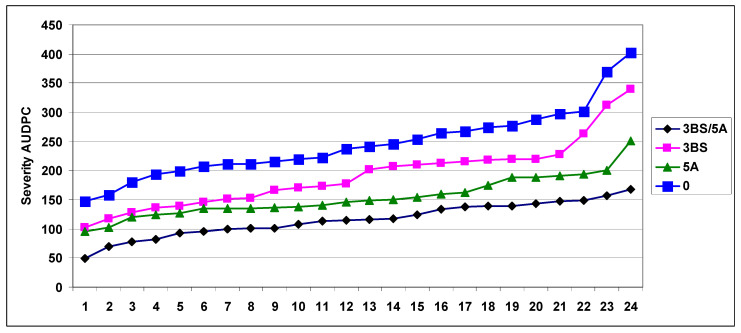
Area under disease progress curve (AUDPC) values for disease severity in the CM82036/Remus population across years, isolates, and location, 2002–2003. Limit of significant difference (LSD) 5% for genotypes 32.54, for quantitative trait locus (QTL) groups 6.62 [53].

Mesterhazy et al. [49,53] tested the CM82036/Remus mapping population developed by Buerstmayr et al. [50] to map Type I and Type II resistance. Spray inoculation was used in both experimental sites, Szeged (Hungary) and Tulln (Austria), in 2002/2003. For the test, 24-24 genotypes were selected without QTL, 3BS, 5A, and 3BS+5A together. Two-two isolates of *F. graminearum*, *F. culmorum*, and *F. avenaceum* were used, accompanied by a *F. sporotrichioides* and *Gerlachia nivalis* (Syn. *Microdochium nivale*, *F. nivale*). Using the spraying inoculation, it was possible to know the genetic background of the QTLs and to see their rate within the total resistances suggested by Ruckenbauer and Buerstmayr, who published the presence of the two QTLs in this mapping population [54,55]. The disease severity index presented clear differences between genotype groups. 5AS (AUDPC 153) (Type I) is stronger than 3BS (AUDPC 192) (Type II), their combination gives the least severe data (AUDPC = 116), and the No QTL group showed the highest severity (AUDPC = 245). The reduction compared to the No QTL group was 22% for the 3BS, 37% for the 5A, and 53% for the combined QTL group (Figure 6).

**Figure 7:**
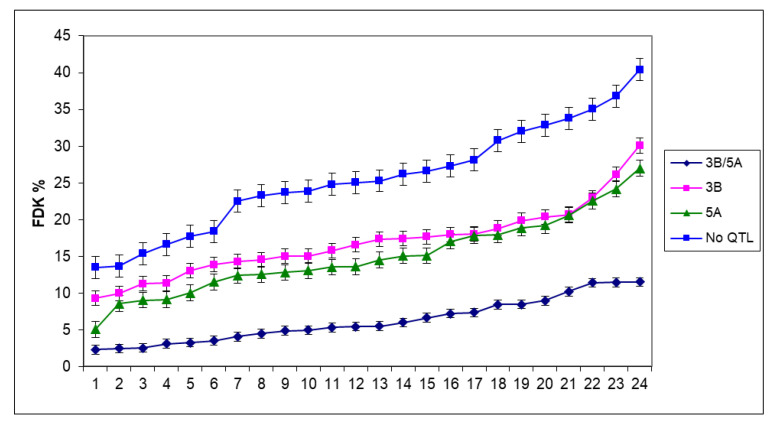
FDK values (percentage) in the CM82036/Remus population across years, locations, and isolates, 2002–2003. LSD 5% for genotypes 3.37, for QTL groups 0.67 [53].

It seems that the variability within the group was significant, so we can assume the presence of other QTLs in the population. It is suggested to estimate the real genetic value of the QTLs is the mean product of the four most susceptible lines, as counted in the breeding work, and the probability to have other unidentified QTLs was much lower here. For FDK, the 5A was also stronger than the 3BS, but the difference was smaller and not significant (Figure 7). However, the combined effect of the two QTLs on FDK was significantly more effective than was found for the visual symptoms, and the distance was also larger between the No QTL group and the other variants. The mean reduction was 41% for 5A, 34% for 3BS, and 76% for the combined QTL group. The ANOVA of the FDK data (Table 2) shows that the main effect of genotype significantly differed from the otherwise significant two-way interaction containing a genotype component, and this was valid for the three-way interaction, where only one of the three was significant. It seems that the genotype effect is dominant and the resistance ranking is rather similar under different conditions. The variability within groups was the same for visual scores. 

**Figure 8:**
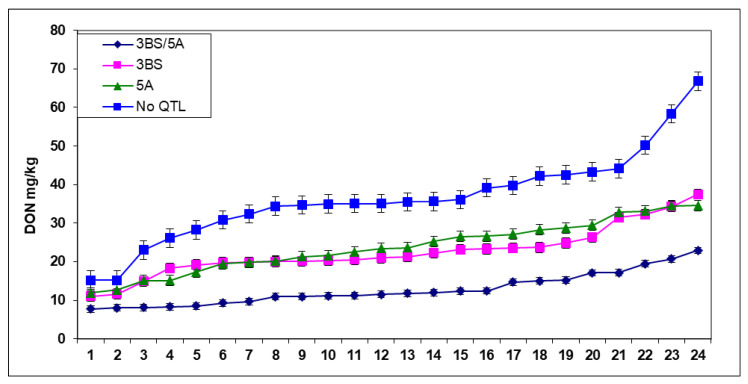
DON content (mg/kg) in the CM82036/Remus population across years, locations, and *F. graminearum* and *F. culmorum* isolates, 2002–2003. LSD 5% for genotypes 3.37, for QTL groups 2.16 [53].

For DON, the situation is similar (Figure 8). Only the four DON-producing isolates were considered, and the reduction compared to the No QTL group was 23% for 5A, 22% for 3BS, and 65% for the 3BS+5A together. As 3BS was significantly less effective in controlling visual symptoms than 5A, in FDK, they were much closer to each other with no significant difference between them. The mean for DON in 3BS plants became slightly better (without significant difference) and in 11 cases of the 24, the 3BS plants were slightly lower in DON contamination. The improvement can be explained by the enzyme glycosyltransferase mediating the detoxification of DON to the non-toxic DON-3-glucoside [56].

This means two things: the effects of Type II resistance can be detected without any problems from the spraying inoculation, and the 5A is really an effective QTL for FHB, especially when we see its DON performance as confirmed by Steiner et al. [57]. In all traits, there is a transgressive segregation of the two effective QTLs work as described by Liu and Wang [44] for visual symptoms. 

**Figure 9:**
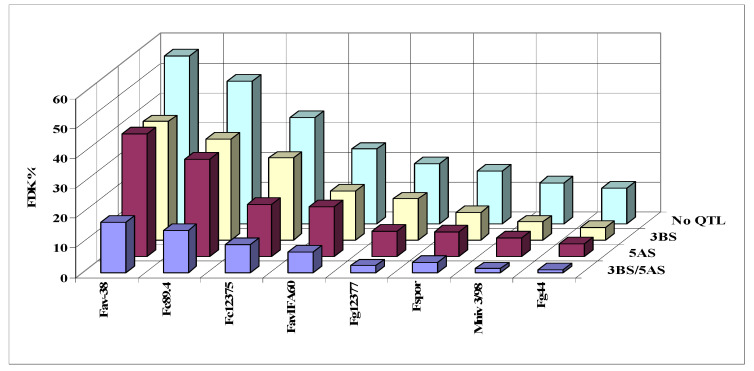
Resistance protection of QTLs 3BS and 5AS against eight isolates of Fusarium on the CM82036/Remus population in wheat, Szeged-Tulln, 2002–2003. Fav: *F. avenaceum*, Fc: *F. culmorum*, Fg: *F. graminearum*, Fspor: *F. sporotrichioides* and Mniv: *Microdochium nivale*, *F. nivale*, *Gerlachia nivalis* [48].

In all countries, several *Fusarium* species are responsible for the FHB. In Hungary, *F. graminearum* is the ruling species, but *F. culmorum*, *F. avenaceum*, *F. sporotrichioides*, and *F. poae* can occur in given years and areas [58]. In Belgium, *F. graminearum* and *F. culmorum* dominate, *F. avenaceum* and *F. poae* can have importance [59]. Poland has *F. graminearum* and *F. culmorum* as main pathogens and *F. crookwellense* occurs also at higher density [60]. In Germany, *F. poae*, *F. avenaveum*, *F. culmorum*, and *F. graminearum* have higher significance [61]. From Canada, *F. poae*, *F. graminearum*, *F. culmorum*, and *F. avenaceum* were identified as main pathogens [62]. So, the multiple exposition of the wheat to different *Fusarium* spp. is a general fact. In this experiment [53], eight isolates from five important *Fusarium spp*. were tested. Figure 9 shows the QTL effect on the infection by the eight isolates. The 3BS and 5A QTL have very similar effect to all of them; therefore, the QTLs inherit a complex resistance to the different *Fusarium spp*. The results are very similar to what we had in a test with genotypes of different origin against different *Fusarium spp*. Other papers [3,35,63] supported the non-specificity also. Here, we got the molecular genetic explanation, too. *Therefore, we can speak about a common, species-non-specific resistance in wheat for the Fusarium spp. and the two QTLs tested. As a conclusion, this work should be extended including more QTLs and the not tested Fusarium spp. The Fusarium population can change sharply between years and in different regions, other Fusarium spp. are dominant. Therefore, the breeder needs information whether his problem can be solved against F. langsethii or other species that are important in his area where he/she has F. graminearum resistance for sure. It is a fascinating theoretical challenge, but also a practical breeding problem. How our resistant cultivars will behave to the different Fusarium species structure in regions where not these two now known QTL are used. The experimental hypothesis is that probably yes.* This is supported by the observations that breeding material originated from other continents not having these two QTLs are mostly useful in the recipient countries. Nevertheless, we did not have experimental proof for it. The only one paper that compares resistance from genetically very different winter wheat cultivars show highly similarity in resistance behavior to different *Fusarium graminearum* and its stricto senso species and also in *F culmorum* isolates from large geographical distances [64].

The experimental results showed further that the spraying inoculation does not show clear Type I resistance, but both Type I and Type II together. Bai and Sharen [65] and Zwart et al. [66] showed similar results. Their test gave further information. The resistance of 5AS and *fhb1* plants (in sensu Buerstmayr et al. [9] was similar to the difference mentioned earlier. This QTL (*fhb1/QFhs.ndsu-3BSi*) was not found in the European winter wheat materials tested. However, the plants having both QTLs had only half of DI, FDK, or DON, confirming the transgressive segregation described for Sumai 3 [44]. Arruda et al. [67] worked with spawn inoculation that was a natural close variant of the spray inoculation, and the effect on the 3B chromosome (*Fhb1* QTL) with its Type II resistance could be verified, supporting the overall view of resistance of the spraying inoculation. As *fhb1* is only responsible for half of the resistance in the plant, it is not an accident that the breeding focusing on the *fhb1* QTL could not produce a large amount of resistant plants. In my opinion, using Sumai-3 and working with point inoculation, the 5AS QTL could not be found, and when it was there and helped to secure high resistance, it was thought to be a function of the *fhb1* QTL. Steiner et al. [57], working with the same population we did (CM82036/Remus), found another QTL *Qfhs.ifa*-*5Ac* in 5AS, which is responsible for resistance to FHB and anther extrusion that could be interesting for the future. Spraying inoculation was used, and visual head symptoms were rated. However, no influence on DON contamination was tested. A new development in China is testing the combination of the Type I and Type II resistance by spraying inoculation [68].

It should also be considered that Type 1 ought to be characterized because it does not contain the spreading function. For this reason, incidence (rate of infected plants without looking at the severity of the infection) was evaluated. In addition, severity was evaluated, indicating the rate of the infected spikelets of the plants showing infection (incidence) [9]. Most authors made several ratings to follow disease development. Usually, an AUDPC or mean of ratings was calculated. However, this automatically contained the spreading function. This is true also for the evaluations, made on the 21st day after inoculation [47], as the symptoms summarized the disease development until this date and the spreading function is clearly behind the numbers evaluated. Reminiscing on past decades, this is not a surprise, as this is also true for the disease index that has the same background, as this also contains the multiplication of the incidence and severity. This explains why the spraying inoculation covers both Type I and Type II resistances and the original Type I cannot be presented purely from the response to spraying inoculation. Type II has a clear definition and this term should be kept for the future, as we have no reason to change it. The real difference between the types is that Type II only considers the disease progress from the inoculated ovarium (point inoculation) to both ends of the head. It is clear that the infection starts in this case from the ovary and neighboring area. It does not consider other traits influencing infection from the inoculation site on the outer surface of the head, its complicated way to the ovary, the possible role of the lemma, palea and head axis, the role of anthers (anther extrusion or anther retention, etc.), are not considered, see more details at [69,70]. Of course, this is also a time function, it takes time for a conidium landing on the palea, germinate, and grow into the inner area of the floret. As many parts are unknown, the research is tasked to better understand the process. There are many other phenomena we now know also influence disease development, together or independently from the spreading function. There are antifungal proteins, secondary metabolic products that may influence disease development, morphology traits, histology, and physiology of the host and pathogen [71].

As both components have the spreading function, it is not an accident that corresponding FDK and DON values accompany both. This corresponds to the finding of Schroeder and Christensen [27]. For this reason, we have to think about the meaning of the data we classified as Type I resistance data for the last decades. All these can be summarized in terms of the total resistance in Type I. This can be dissected to Type II, III, IV, and V and others like another extrusion etc. could be added when justified. In each genotype, there is a different mix. They can be evaluated by the spraying inoculation and its different components can be identified by a further evaluation of the harvested grains (FDK, DON, etc.), or other specific inoculation methods that could be used. For Type II, a separate test will be needed. When we see their biological role, we return to the *resistance component* term we suggested earlier [34]. This describes the real situation better than the resistance type that indicates their fully independent presence in the plant. As a result, the scientific logic would also be kept, the most important comes of which comes to the forefront. I have chosen "total" as Liu et al. [72] used the “overall” word for resistance being active in the seedling and adult stages at the same time. We have found, among plants selected for seedling resistance, genotypes with good FHB resistance, but this was only a tendency, not a general rule we could use in a general breeding program [52]. In several cases [28], both spray and point inoculation were used, but it is difficult to say how much percentage of the total resistance is explained by Type I and II. When we have only spraying or other natural close inoculation (spawn), it is unknown, in agreement with Dill-Macky [23], how much of the identified resistance is caused by the not tested Type II QTLs. We know now that QTL *fhb1* (Type II) determines alone maximally a medium or a somewhat higher resistance securing about 50% of the total resistance in CM82036. The same is true for 5AS QTL [44]. However, their very effective cooperation can secure a high resistance level. As they also secure low FDK and low DON at a much higher level than the two QTLs alone, it is not an accident that they are popular in breeding programs. During QTL analyses, genotypes were found with nearly pure Type I QTLs like in Frontana [28], in CM82036 the rate can be 50% or in others even higher.

Most of the breeding firms use some form of spraying inoculation or natural close spawn inoculation for total resistance. Scientific research mostly uses Type II resistance. Additionally, in most cases, only visual symptoms are mapped. However, these define only about 50% of the resistance of Sumai 3 (or its derivative CM82036) and this can be much higher and or lower in others. From Figure 8, it is clear that these two QTLs similarly protect against four *Fusarium spp*. and *M. nivalis* [53], e.g., the resistance seems to be also non-species-specific. In other genotypes, the rate of the two resistance types is mostly unknown. Here, I would like to cite Schroeder and Christensen [27] again who insisted that the Type I and Type II resistances could be differentiated only by their reaction to FDK. This is not obtainable presently. Based on current research, it is not certain that the FDK has such a differentiation power between the two resistance components. What is really happening is that at the point inoculation, we have a resistance-dependent slower or more rapid extension of the infection differing in time, and the top and the base of the head the grain are normally later and less infected and may remain healthy. In the spraying and natural inoculation, the multiple infections can be profound within a head; therefore, the rate of infected scabby heads comes mostly from the more or less uniform infection of the spraying infection. The DON contamination mirrors these processes well. For this reason, I think that Schroeder and Christensen [27] did not recognize the real role of the FDK, but they were the first to stress its role in resistance to FHB, which was not considered seriously by the later generations. It seems that a reliable rate of the resistance components can be identified correctly when a mapping population plants is selected that has only Component I and Component II plants. Then, following a spraying inoculation, we can see the weight of the components in the given genotype, as it was done in the CM82036/Remus population [53]. In the chapter “Breeding aspects”, we will come back to this problem from the practical point of view of the breeders, and in the chapter "QTL parts", there are also important new insights that might influence further developments.

As a conclusion, without Type I resistance component, the securing of high level resistance is questionable. 

## 4. Resistance to DON (Component/Type III) and FDK (Component/Type IV)

Lemmens et al. [24] reported that the ultimate goal of breeding is to decrease mycotoxin contamination, but generally, toxin contamination is not measured, because it is costly. Therefore, breeding concentrates on the visual symptoms, on ears, and much less on FDK, and only the advanced lines will be monitored for mycotoxin contamination, if at all. The result is that we do not know how the basic relations work in the populations, which are the sources of resistance that decrease not only visual symptoms, but also mycotoxins. The resistance to DON is a complex trait with several background mechanisms [3,34]. It can be caused by a toxin degradation mechanism reported by Miller and Arnison [73]; the increased resistance can cause less disease, so this does not require a separate resistance trait. However, we have cases where toxin contamination is consequently less than forecasted based on symptoms over the years. This can be a result of detoxification of DON to DON-3 glycoside [24,56,74]. The overproduction of DON is a fact [26], but the genetic background is unknown. 

**Figure 10:**
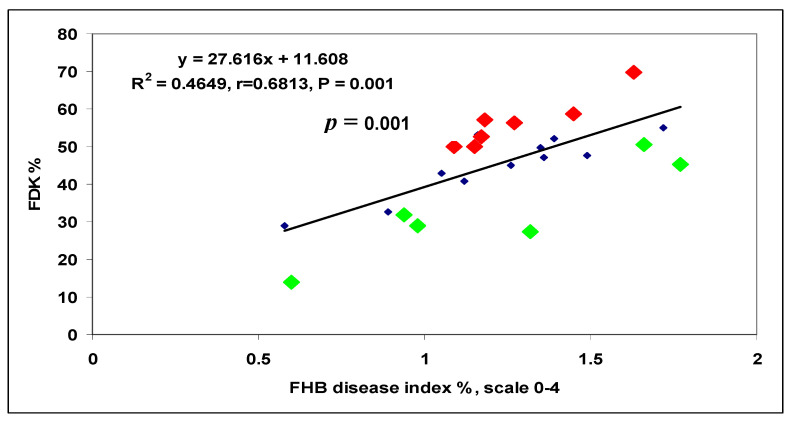
FDK overproduction and resistance in FHB resistance tests in winter wheat genotypes against two *F. graminearum* and two *F. culmorum* strains, 1983–1988. Red data points: FDK overproduction, data from the regression line are farer than the LSD 5% 4.49 to the larger DON concentrations; green data: resistance to kernel infection, data from the regression line are farer than the LSD 5% 4.49 to the lower DON concentrations. LSD 5% for FDK is 4.49 [3].

Mesterhazy [3] described three further resistance types. For five years (1983–1988), four isolates were used independently in three replicates; altogether, 60 replicates were involved. This secures the reliable data. He [3] reported the resistance to kernel infection (FDK), describing the lower FDK level in one genotype compared to another genotype not differing significantly from the other in visual symptom severity (DI), termed Type III resistance (Figure 10). Here, seven genotypes were toxin overproducers, six had kernel resistance, and nine responded regularly. Similar findings were found in [34,35,40]. However, it was realized [26], that later on not only can an FDK decrease identified, but extra FDK overproduction also has significance for breeding, and resistance to DON was also mentioned and confirmed [34,35]. 

The statistical method [3] was modified. It was argued earlier that we can prove resistance to kernel infection when the two genotypes do not differ significantly in resistance (e.g., disease index), but show significant differences in FDK. The newer evaluations [26] are stricter. The distance for all data points was calculated between them and their corresponding point on the regression line. We have the LSD 5% value for the FDK. When the difference between this point on the line and the data point is larger than the LSD 5% value, we can speak about proven relative resistance to kernel infection or a significant overproduction of the scabby grains. This applies also to DON. Additionally, this identifies overproduction or relative resistance, when no genotype within significant distance exists on the *x*-axis. This is also an advantage. In the 1990–1993 tests, six genotypes (30%) showed FDK overproduction and six (30%) relative FDK resistance in tests with four isolates when compared to DI data, and eight responded proportionally for the traits (40%). The DI/DON comparison resulted in four DON overproducers (20%) and 7 genotypes (35%), with relative DON resistance of the 20 genotypes tested. When FDK and DON were compared, four DON overproducers (20%) and only 3 relative DON resistant genotypes could be identified (15%) and 13 (65%) responded proportionally with FDK. As correlation was between DI/FDK r = 0.74 (*p* = 0.001), for DI/FDK r = 0.57 (*p* = 0.01), and for FDK/DON r = 0.73 (*p* = 0.001), it is clear that the FDK is a better trait than DI to estimate DON response. 

**Figure 11:**
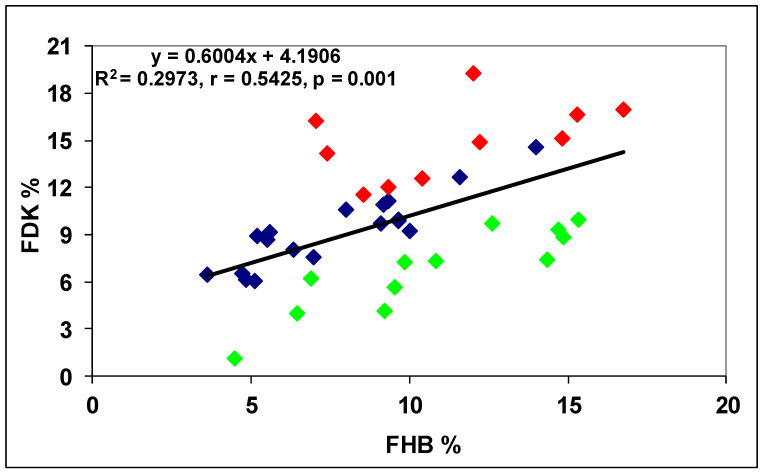
DI and FDK regression in FHB resistance tests in winter wheat genotypes against two *F. graminearum* and two *F. culmorum* strains, 2009–2012. Red data: FDK overproduction, data from the regression line are farer than the LSD 5% 1.66 to the larger DON concentrations; green data: resistance to kernel infection, data from the regression line are farer than the LSD 5% 1.66 to the lower DON concentrations. LSD 5%: for FDK is 1.66 [26].

**Figure 12:**
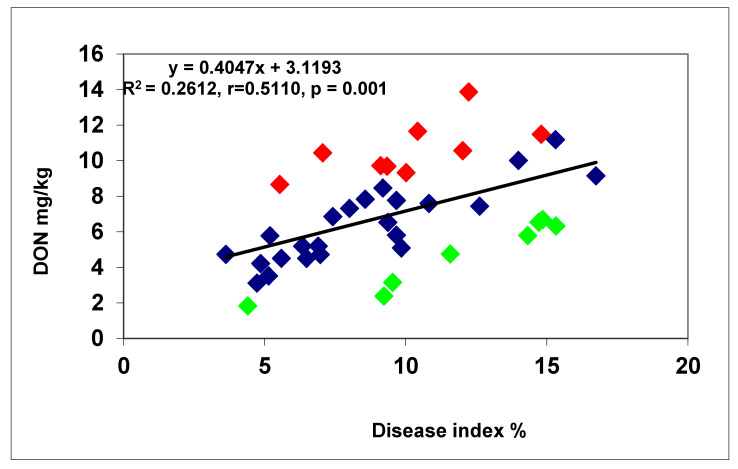
DI and DON regression in FHB resistance tests in winter wheat genotypes against two *F. graminearum* and two *F. culmorum* strains, 2009–2012. Red data: FDK overproduction, data from the regression line are farer than the LSD 5% 2.14 to the larger DON concentrations; green data: resistance to kernel infection, data from the regression line are farer than the LSD 5% 2.14 to the lower DON concentrations. LSD 5%: for DON is 2.14 [26].

**Figure 13:**
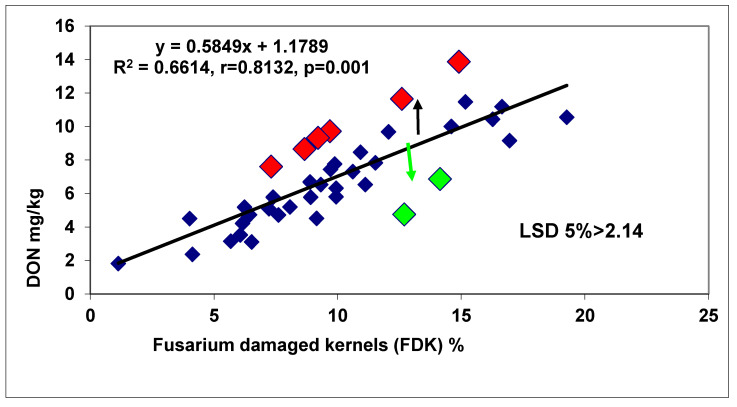
FDK and DON regression in FHB resistance tests in winter wheat genotypes against two *F. graminearum* and two *F. culmorum* strains, 2009–2012. Red data: FDK overproduction, data from the regression line are farer than the LSD 5% 2.14 to the larger DON concentrations; green data: resistance to kernel infection, data from the regression line are farer than the LSD 5% 2.14 to the lower DON concentrations. LSD 5%: for DON is 2.14 [26].

In another test, 40 genotypes were tested [26]. In this test, we had 96 replicates behind each data point; it became possible to prove the overproduction and DON resistance occurred at a high probability. From data with several replicates, the LSD 5% values are normally too high to use them for such an analysis. We found a correlation between DI and FDK data, r = 0.54, *p* = 0.001 (Figure 11). Ten genotypes (25%) showed higher FDK severity than the LSD 5% difference of 1.66 between genotypes, e.g., an increased susceptibility to FDK, and 12 (30%) produced less FDK than forecasted by the proportional response. These were the relative FDK resistant genotypes and 45% produced DON proportionally with the FDK data. The correlation between DI and DON was r = 0.5425, *p* = 0.001 at n = 40. For the DI/DON relation 9 (22.5%), DON overproducers and seven (17.5%) relative DON resistant genotypes were identified. Twenty-four (60%) genotypes belonged to the group that did not differ significantly from their data points on the regression line (Figure 12). The DI/FDK correlation was only r = 0.5110 (*p* = 0.01). The FDK/DON regression was much closer, r = 0.8132, *p* = 0.001 (Figure 12). We had six proven overproducers and only two genotypes with relative DON resistance. Compared to the DI/DON relations, this seems to be a more solid situation (Figure 13). Of the 40 genotypes, nine had for DON overproduction, but in food safety, they can be critical and 12 presented relative DON resistance, so 21 were off types, which is considerable. 

As we did not find it in the literature data, we tested large-scale breeding material to see what these correlations look like. The test was made in two replicates and with four isolates [75]. In Table 3, we show the five years of correlations (2011–2015). 

It seems clear that the disease index provides, in most cases, a significantly looser correlation with DON than FDK. It is also clear that the disease index and DON showed a moderate relationship. The data proves that, in FDK, special additional genetic factors are active to modify the FDK and DON response in an overproduction or to a relative resistance. These latter two were described as FDK and DON underproduction or relative resistance components [3]. The occurrence of the toxin overproduction or higher FDK severity can take 10–15% of the population. The same is valid for the relative resistance, e.g., resistance to FDK and resistance to DON. In some experiments, the correlations between DI and DON can be on the same level, but this is not regular.

The data gave further support to the idea that the disease index is significantly less suitable to forecast DON response than the FDK ratio. The pattern is similar to that in the previous experiments. On the other hand, Arruda et al. [76] compared the usefulness of the trait incidence, severity, disease index, FDK, and DON using the spawn method of inoculation and came to the conclusion that the best traits for resistance were FDK and DON, which agrees well with our experience. However, Arruda et al. [67] concluded that most traits had very low frequency and they hoped to select better combinations with favorable alleles. He et al. [77,78] found useful correlations between DI and DON, but FDK was not tested, so we cannot compare it with the FDK response. It is important that before the genotypes were included in crosses. They were tested for three years for FHB resistance. This is highly important information supporting the line of research we follow.

The analysis showed clearly that the resistance components are well characterized. This is valid also for the FDK and DON overproduction. This is not new, but the real new development is finding their role in the genetic and breeding system. Beside this, the detection of the DON overproduction in 10–20% of the genotypes tested is also an important step to identify new food safety risks beyond those we knew earlier. Therefore, the cultivars must be checked for this trait. This is true also for the FDK, the same ratio of genotypes may show agreeable DI, but bad FDK data. In our praxis, about 50% of the lines having low to moderate DI have high or very high FDK level. The visual rating finishes 21–24 days after inoculation. Thereafter, disease development can be continued in the remaining time of 2–3 weeks of harvest. In a dry season, from flowering to 3–4 weeks, no rain is measured and the morning dew is rare, the natural infection is low or absent, and correlations between traits are closer when artificial inoculation is made. Following inoculation, larger rains come (above 5 mm per day) in 2–3 weeks, an additional disease development will start, but will be realized only later than 21–26 days after inoculation when we stop reading, as the ripening heads cannot be evaluated precisely. In this case, the additional disease progress will increase FDK, but differently at the genotypes. We discovered from Jones and Mirocha [79] that the scabby grains have the highest DON contamination. 

There is another phenomenon, to have DON (normally not a high amount) on grains without visual head infection. DON can be translocated from stem base infection [80]. DON translocation is possible to cells in head not infected by *F. culmorum* [81]. The chaff can also be a source of DON translocated to otherwise healthy grains in the susceptible genotype, but in resistant genotype, the DON translocation was inhibited at little fungal colonization. Further, DON starts to be produced 2–3 days after inoculation [82] in susceptible genotype, but in Sumai 3, did not respond positively. Savard et al. [83] found four days after inoculation 1200 mg/kg in the inoculation point and 500–600 mg/kg in the spikelet below the inoculation point. As in susceptible plants, 6–12 days are necessary to see symptoms, a significant amount of DON can be present at the appearance of the symptoms. It is important that DON translocation seems to be resistance dependent. It was observed at FDK screening that among grains, for example 50 grains, showed characteristic scabby symptoms, however, 10–20% looked normal, only the grain surface showed a light powdery impression. DON was calculated for both the scabby and the scabby+powdery (total) FDK data and the correlation was stronger by 0.1–0.15 with the FDK, including also the powdery grains. This means that the not characteristic scabby grains may also contain DON (Mesterhazy unpublished). 

## 5. Tolerance to FHB (Component/Type V)

The tolerance measures the yield response to FHB infection [3,34]. It is no longer in use, as it is very laborious to detect. We see now that it can be a useful trait, but in normal breeding praxis, it has moderate significance. We remark that yield loss comes from three different sources. 

**Figure 14:**
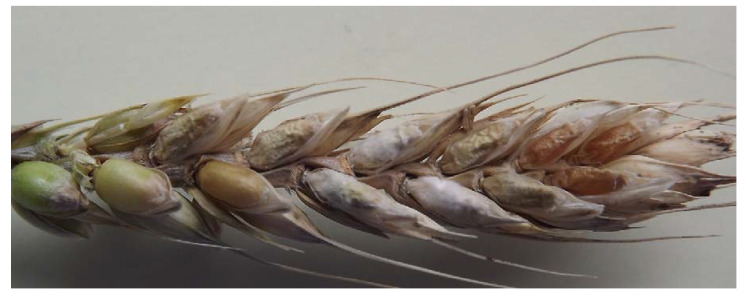
Ear of a highly susceptible genotype. At the bottom, normal green-colored, full sized grain, even the head axis is damaged to some extent. One spikelet higher, the grain starts to show early ripening discoloration, but it is still full size. The next spikelet is infected, the green color covered by mycelium, and shriveled. The next spikelet is light brown close to waxy ripening. Thereafter, there are four strongly infected spikelets with massive mycelium coverage, then, two infected grains similar to the second one from the bottom, and at the top three strongly shriveled, but bleached normal colored, not directly infected, grains.

**Figure 15:**
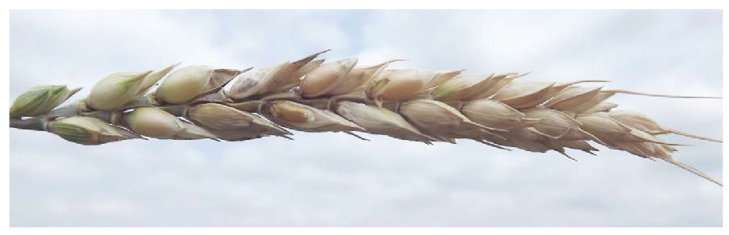
A medium resistant genotype with one spikelet infected, and above the infection site, normal size grains sit in the spikelet without shriveling (except the spikelet above the infected one) and only the light brown discoloration shows the accelerated ripening process. From this head, we will have a yield close to the full healthy heads.

The first is the decrease in grain number/head, which can be up to 70–80% in extremely susceptible genotypes. The second source is the rate of light scabby grains that are blown out and fall to the soil. In experiments with hand harvesting and careful threshing, this is not a problem. The third reason is the bleaching; there are dead spikelets that are indirectly infected, but the infection of the head axis stops the metabolism and the grains become smaller with normal color. They are responsible for the shriveled and normal colored grains at the top of the head (Figure 14). Multiple infections in an ear are often the case in susceptible cultivars; they may originate from different dates that can make evaluation harder, as described by Dill-Macky [23]. Using a more resistant cultivar, the assimilate transport can function at a lower rate, but the consequences on the grain shriveling is significantly smaller (Figure 15). These three phenomena explain the tolerance or differing yield responses. We cannot exclude the presence of further agents like fertilization, other diseases, draught, water stress, etc. [71,84]; therefore, research in this field would be useful. 

In conclusion, the resistance components are valid, well-defined traits. In a larger part of the population, the responses to DI, FDK, and DON agree well; however in a smaller, but significant part of the population, the FDK, DON, and DI reactions can be very different. Therefore, more attention is needed to manage a well working selection system than only a screening for DI. Of course, it is assumed that the diverging responses are genetically regulated. Therefore, the genetic background should also be better understood.

## 6. QTLs and Their Value

Here, references were collected that mapped more traits on the given mapping population. However, first an important methodical problem should be discussed. In the mapping populations, usually wide flowering time differences exist and the artificial inoculations should adapt to it (11–18 days, 4–5 inoculations in a season in field tests). This might cause problems as the weather normally changes over such a long period. In several papers, we see that QTLs for flowering date can influence FHB data [9,85]. Table 4 shows the DI and FDK means for the different inoculation days in 2002 and 2004 of the Frontana/Remus population [86]. In 2002, the earliest inoculation gave the highest infection, both for AUDPC and FDK. Thereafter, a slow decrease was seen in DI, and the FDK reduced to half at the end of the readings. The same population gave perfectly different numbers in 2004: the AUDPC increased from 151 to 832, but the FDK remained stable. When there are two years with weather conditions similar to each other, like in 2004, and only AUDPC, there is a decisive effect of flowering time. It could be the opposite. When high infection starts at the beginning because of an early warm and wet period, and at the end (during cooler weather), much lower data are yielded. In these cases, we observed significant flowering time effects, but in opposite directions, depending on the weather. This caused deviations that cannot be corrected. Additionally, a significant part will not have genotype-related phenotyping and QTLs from this data set will be high percentage artifacts. For FDK, there was a definite decrease in 2002 at the later inoculations, but a rather stable performance in 2004. Considering that, the resistance level in each flowering group was nearly the same as there was no significant difference between two inoculations and group means and the minimum and maximum values were close to each other, the data can be pooled into one group. In this case, according to FDK, the whole population can be treated as a pooled material that is impossible based on DI data. As this is not considered in the analyses, QTLs for resistance may be artifact in most cases when a QTL will be identified for flowering time. In reality, this is the influence of disease response caused by environmental traits and not a resistance trait itself. This means that flowering period can significantly influence resistance ranking, and therefore, it can cause a serious error in the QTL analyses following the incomplete phenotyping. This means that the inhomogeneity of the population is a real risk factor.

Steiner et al. [28] (2004) worked with the same Frontana/Remus population that Szabo-Hever et al. [86] (2012) worked with and did not map FDK, and no other authors working with different Frontana populations have such data [48,87], therefore, a comparison for FDK was not possible. Burlakoti et al. [88] (2010) tested Frontana together with Alsen in AUDPC for disease severity and they were found to be equal. In FDK, Frontana was higher, but the difference was not significant. DON in Alsen was 50% less than Frontana, 7.90 versus 18.15 mg/kg, respectively. This is important as very similar ear blight data may result in very different DON contamination. This means that visual symptoms alone were not enough to choose a resistance donor or select against DON contamination. They tested 3BS and 6A (Type II) and 5a (Type I) from the cross Frontana/W9207//Alsen, but from the data, it is not clear how much the two QTL types contributed to the resistance. Alsen was the more resistant parent, but no line differed significantly from it with somewhat better resistance except line 3545 for AUDPC. On the other side, the more susceptible crossing partners did not inhibit to select lines at the resistance level of Alsen.

In the Frontana/Remus population, six experiments were conducted over four years. In two years, 1-1 isolates were used and in another two years, 2-2 isolates were used [86]. FDK was also mapped beside the disease index. Not all QTL were significant in each year and each experiment. In the 6 experiments, 10 markers were found, therefore, 60 possible significant cases could be identified. The marker Xgwm120-Xs12m19_9 was significant only in the first test for DI and this was also the case for FDK, but not for the others. Xgwm526 was significant only in one test of six for 3A, and one of six for FDK, both in Exp. 5. Altogether 12 significant cases of 60 were given for DI and 18 cases for FDK. For some reason(s), FDK seems to be a better trait. When we see the mean performance, we see that *F. culmorum* had only six significant cases for LOD values. For *F. graminearum*, we had only two significant cases. As shown in the paper, the two *F. graminearum* experiments had low DI and FDK data; this can be the reason for the poor QTL result, as differentiation of the genotypes was low. The other finding was that a QTL could have different functions. Comparing the Xgwm526 has not been effective for DI on 2B, but was significant for FDK. The 3A QTL was effective only for DI, but ineffective against FDK. The 3D was ineffective against DI, but had a significant influence on FDK. In 4A, we had a significant effect on DI, but no influence on FDK. 4B was significant for both traits. 5A was specific only for FDK. 6B outlined a significant effect only in FDK, and 7B was effective for both traits. In summary, of the ten QTL 3 that were effective for both DI and FDK. Three were specific to DI and four influenced only FDK. Several QTLs overlapped with the results of Steiner et al. [28], but there is no good explanation for the QTL on 7B that was strong in our tests, but could not be identified in Tulln. From the genetic variance, 61.6% could be explained for DI by eight QTL and for FDK, 57.7% was explained by seven QTL. 

The lesson for us was that we should increase the number of isolates used, and we should shorten the flowering period of the population. For this reason, a new mapping population, Mini Mano/Frontana was developed [25], with a far higher DH number than 200. The too early and too late genotypes were discarded and several of the very tall plants were omitted. In the test, 168 genotypes were used. Six isolates were used yearly, so 12 experiments served as outgoing materials. The FHB data were checked for normal distribution and five tests were discarded, so seven remained. There was no data transformation. The heading date did not show any significant correlation with DI, so the decrease in inhomogeneity for this trait was successful. The FDK and DON showed significant, but not very close, correlations with heading date (r = 0.5 and r = 0.38). The taller plants tended to have higher lodging values (r = 0.68, *p* = 0.001). Therefore, it seems that beyond flowering time, the omission of the very tall and dwarf plants could reduce the inhomogeneity of the population and switch off traits that strongly influence FHB development. The DI/DON correlation was r = 0.319, the FDK/DON r = 0.54, both being significant at *p* = 0.001 but the FDK correlated closer with DON data than DI did. 

The mapping results across the seven tests yielded significant improvement compared with other tests [48,87] (Table 5). Altogether, 15 QTLs were identified for FHB. Therefore, in the seven tests, we had 105 possible LOD values. Among them, 73 were significant. Six QTLs were significant in all seven experiments, four QTLs had five, four had five QTL, two showed four and 1-1 had 2, and 1 had zero. In FDK, 62 positive cases were found. One QTL was significant in all (7) tests, six had six significant LOD values, one had five LODs, four had three, two had one, and one had zero significant LOD numbers. Compared with the Frontana/Remus population, these numbers are much higher. The size of the significant LOD values was between 2.14 and 6.86 for DI as well as 2.01 and 5.42 for FDK. This refers for the individual experiments, their means in Table 5 shows somewhat higher numbers.

The number of identified QTLs in the Mini Mano/Frontana population doubled [25]. Of them, 15 QTL gave positive responses for some of the traits. Two QTLs were active only for DI. Seven acted for DI and FDK, but these nine QTL did not have any influence on the DON contamination. The QTL 3A is not listed here, as it had no measurable response for DI and FDK, but it gave a positive answer for DON. We detected only six of the 15 QTLs influencing DON contamination. Of them, only four had a reducing capacity for all three traits. This shows clearly that the effect of the QTLs is strongly structured, and without knowing the influence of the given QTL for FDK and DON, the breeding value of the given QTL cannot be evaluated. We should mention that for DI, two QTLs explained more than 20% of the variation and sic counted values between 10 and 19. For FDK, one gave more than 20%, and eight gave numbers between 10 and 20. For DON only six QTLs were found and three were between 10 and 20%. 

When comparing the LOD values for Frontana for the identical QTLs in [25,28,86], we saw rather large differences. We do not think that the QTLs were here 2–3 fold stronger in Mini Mano/Frontana. The variance explained (VE) above and near 20% means only that the background noise was smaller, and this allowed the detection of double the amount of QTLs than were previously found. The other feature is that the number of identified QTL is about double than that mentioned in other papers [28,48,87]. This means that the traditional mapping work sees about 50% of the QTLs, only those that could be detected. When we see the correlations between plant height, lodging, and *Fusarium* traits, further improvement in the increasing reliability of the QTL analysis is possible when the tall and mostly lodging-susceptible genotypes do not influence the FHB response to different traits. 

Besides these results, other authors detected structured QTLs with different functions. Lemes da Silva [89] identified eight QTLs; the Qksu.fhbE-7AL influenced only FDK in one year. The *Qksu.fhbE-6BS* determined AUDPC only for one year, the *Qksu.fhbE-5AS*, the FDK and DON, and the *Qksu.fhbE-4BL* determined only visual symptoms. The *Qksu.fhbE-3DS* determined DON and 1000 kernel mass, the *Qksu.fhb-2AS*, AUDPC, and FDK, the *Qksu.fhbE-1BS* determined the visual symptom development, DON, FDK, thousand corm weight (TKW), and the *Qksu.fhbC-1AS* influenced visual scores, DON, and FDK. The combinations found by Szabó-Hever et al. [25] were quite similar. Most QTLs explained less than 10% of the variability, but in eight cases, the values were higher with the maximum of 20% for TKW. Liu et al. [90] found on 4BS a QTL determining FDK, severity, incidence, FDK, and DON. The 3BL coded severity, FDK, and DON, and on 4DS incidence, severity, FDK, and DON, but only incidence was positive for DI. Petersen et al. [91], also identified QTLs with differing influence on the *Fusarium* resistance traits like visual evaluation, FDK, and DON. The estimation of FDK is critical, and when the shriveled but healthy-looking grains are included, very high numbers can be achieved [78]. These originate from bleached spikelets having no direct infection (see Figure 13 in this paper), but as their tracheas were destroyed and assimilate transfer was stopped by the fungus, the delivery of assimilates and water became impossible. Besides this, FDK and DON data were investigated only for 2012, so the repeatability of the results cannot be checked. 

Arruda et al. [67] mapped all the important FHB traits. They found also that QTLs had different functions, so DON was determined only by two QTL (1D, 3B). Interestingly, incidence was influenced by five different chromosomes (7D, 6A, 4D, 4A, 7A), but disease index was regulated only by 3B. On the other hand, the spawn method has the disadvantage that the inoculation time is not so easy to identify; this can cause instability in the system. The epidemic severity was high, so the differentiation of the genotypes was acceptable. Tessmann et al. [92,93] used spraying inoculation and identified QTLs influencing FDK and DON on chromosomes 4A, 5B, and 6B, and they reported additional FDK and DON specific QTL in several genotypes. Among the breeding lines, they identified highly FHB resistant genotypes with low DON contamination. He et al. [94] found two QTLs for DON; one was independent of FHB resistance, and the other was located as a minor QTL on 3BL chromosome. Wu et al. [95] found that QTLs Type II controlled both DI and DON. This means that we have convincing genetic evidence for the different role of the QTLs. It is also clear that the DON and DI or FDK regulations are not synonymous expressions. Therefore, the skepticism of Ma et al. [22] about the resistance to kernel infection or DON resistance is not supported by novel data. However, we are currently at the beginning of a process of understanding and that should be continued. 

The majority of the QTL work concentrates on the visual symptoms [21,53,57,96,97,98,99,100,101] as shown and many others. They applied spraying or point inoculation and analyzed only the visual symptoms. Haile et al. [102] in their review of durum wheat FHB resistance listed a number of QTL analyses, but no papers were cited where the reaction of DON could be mapped. Low DON contamination in durum wheat is top priority. Most of the early QTL research was the same [9]. QTL *fhb1* was one of the few exceptions [9], and the reducing influence on DI, FDK, and DON is well documented. As in recent years, the QTLs had a much more complex and diverse effect, influencing one or more traits. In our opinion, the QTLs identified for DI, severity, or incidence should be extended to FDK and DON in order to have a higher chance of decreasing DON contamination. For practical breeding, the spraying inoculation is more important, as this signals the commencement of both Type I and Type II resistance, and resistance components for FDK and DON can be evaluated from the data. The best resistance until now was the combination of Type I and II in Sumai 3. It appears that a combination of the QTLs with different functions is the way forward for future research. 

Liu et al. [29] worked with the spawn method, but this is less reliable as inoculation data cannot be secured exactly. Therefore, more epidemic waves can merge. The disease pressure was rather uniform and high over the different years analyzed. Prediction of the FHB resistance depends mostly on the number of markers used, but no further improvement was seen above 1000. 

QTL mapping has been done in two main directions. Populations and genotypes were tested in most cases for Type I and Type II resistance. *As Type I is often mapped as incidence or severity, it would be better to use the disease index in the future.* I think that research should be to compare visual data with FDK and DON data. As mentioned, most of the past and present literature considers only visual symptoms [5,6,9]. This means that in most cases, we do not have an idea about what the influence will be of these QTLs on DON contamination will be. The possible functions of QTLs are presented in Table 6. As most QTLs are characterized by DI (or separately by incidence and severity), their breeding value cannot be determined as the effect against FDK and DON is not known. For this reason, they are not suitable for MAS because the decreasing effect of DON is unknown and may be absent with a large quantity of the QTLs. For the reasons presented, the marker assisted selection is not yet a general tool, it can be applied on several well characterized sources, but the vast majority of the genotypes having unknown resistance to FHB should yet be detected first, then characterized and later used them. The critical point is the validation, e.g., to check the effect of the donor QTLs in different crosses. The other problem is that most of the QTLs are of small effect [103]. It seems that authors overestimate the significance of Type II resistance for the breeding of resistance and underestimate the problems arising from the use of partially characterized small and medium strength QTLs. 

Of course, the measurement of these traits and discovering their genetic background has two very important fields. One is the characterization of possible crossing partners and resistance sources, and the qualification of the product of the breeding process. The variety should not only have DI resistance, but should also secure low DON contamination. For these tasks, improved methodology is necessary; otherwise, we might produce artifacts that do not benefit the research. 

## 7. Breeding Aspects

The reality is that the QTLs have different functions. As QTL analyses were made in spraying inoculations and point inoculations, their results cannot be compared. What seems to be certain is that the traditional Type I resistance is significantly more effective, as anticipated, and the response to spraying inoculation contains the effect of the Type II resistance. This means that the spraying inoculation is more important for breeding than generally estimated. Actually, this is what most breeders are doing; they use some version of this method. The spawn method is popular in the US [67,76]. It can be considered as a variant of the spraying inoculation being more natural but less reliable. For this reason, Type II resistance will lose in significant resistance breeding, but its significance remains when the resistance background should be characterized. The US experience in the year 2000 and thereafter showed that the *fhb1* resistance did not solve the problem and the Chinese progress was slower than expected. For this reason, the combination of Type II resistance, which is part of the total resistance and the Type I resistance measured by the response to spraying inoculation, provides a more successful breeding philosophy. Mostly incidence, severity, or their combination disease index are measured, and they may be differently regulated. Additionally, the visual symptoms are not enough to characterize the effect of the resistance, their relationship with the FDK and DON contamination is also of high importance. Spraying inoculation has been successful [104] and highly resistant genotypes were created with this philosophy, so it is a good option for practical breeding.

For us, those QTLs are important that, additionally to DI, reduce both FDK and DON. In the Mini Mano/Frontana mapping population, only six of the 15 QTLs had influence on DON. Many papers published similar results indicating that the Frontana story was not an exception. For this reason, genetic research should use the best methodology, and the selection and control of crossing partners as well as the registration trials need similar accuracy. 

In the practical breeding, the case is simpler. He et al. [77] used spraying inoculation at CIMMYT, evaluated disease index, and DON control was conducted on selected elite lines in the 2nd and 3rd year. In an unknown material, as most winter wheat nurseries are not known for QTLs, a screening of the nursery is important. After having considerable information, the breeding program can be modified to cross only partners with moderate or higher resistance to FHB. Then, several heads can be inoculated by spray inoculation by one aggressive inoculum and a negative selection can be done by also considering other traits. In F_2_ and F_3_ generations, no artificial inoculation was necessary when parents were checked; otherwise, the one row from the ear-to-rows of a combination can be inoculated by FHB. The best homogeneous lines from ear-to-row lines coming to the B lines in 5 m^2^ will be tested without replicates for yield and other traits, and one row 1.5 m long will be exposed to artificial mass inoculation using the same method as mentioned earlier [75]. A negative selection follows again and the supposed plus variants should be checked for FDK. The selected plus variants (C lines, n = about 250–300) tested in four replicated trials should be checked by more isolates for resistance. We used four, (three should be the minimum) in one replicate in two row plots as described earlier with spraying inoculation and humidity secured by polyethylene (PE) bags. This was the first DON measurement. Ten percent of the samples from the yield of the four isolates was separated and then pooled. After fine grinding, only one analysis was made. Of course, it was only for those whose other traits were OK and may be suitable for registration. In the multi-location tests (about 48 lines), two plot replicates were used. For the most important lines, all isolates and replicates were worth testing, while for the others, the two pooled samples of the four inocula was enough, and others were not measured at all. When yield was not top, but DON and FDK were very low, the line was identified as valuable crossing partner and used in crosses. Irrespective of the high complexity of genetic background, we should select for low DI, FDK, and DON. 

Marker-assisted selection (MAS) is a very popular tool suggested to solve the issues in breeding cultivars with higher resistance. The prerequisite of this work is the correct knowledge of the donor genotype to all traits; otherwise, the work may be unsuccessful. With the use of well-characterized QTLs like *fhb1* and several others, it can be successful. However, MAS with partially characterized QTLs is risky. As Lemmens et al. [24] stated, if DON reduction is the ultimate goal, only QTLs that are known in this respect ought to be used. This is valid also for the genomic selection [105]. On the other side, many of the existing QTLs are not identified yet and most of the known ones are not validated in crosses. For this reason, careful screening helps to detect new resistance sources in our own material [75], where the effects of the unknown QTLs are also included. As knowledge increases, the breeding program will be more successful.

DON control is further important as the toxin overproducers can be discarded by this method. On the other hand, the knowledge of toxin overproduction is an important scientific problem and its genetic background should be known. The variety candidates, control cultivars, and important competitor varieties are also included in this test, n = about 100, differing each year. It was found [65] that about 50% of the plus variants in the first test year will keep their resistance in the next year. Highly resistant spring wheat sources were used in winter wheat breeding, and now we have such plants. 

The variety registration for FHB remains a central task for the future. Without this, we cannot achieve significant improvement in the wheat fields. At least, medium resistant cultivars can be protected effectively with the most updated fungicides and fungicide technology [37,38]. We should remark that breeding is ahead of the science. Dong et al. [106], He et al. [77], and Murphy et al. [107] screened variety trials for visual symptoms, FDK, and DON, and consequently allowed a more precise and successful identification of low DON content cultivars. It is important that the complexity of the FHB resistance in wheat needs a better understanding as stressed by Ma et al. [45]. I think this is a challenge for science. On the other hand, the consequent screening for all traits can identify new genotypes for mapping and detect new QTL that can be useful for breeding to create new cultivars with higher resistance to disease and toxins. 

## 8. Conclusions

In conclusion, we demonstrated that a better phenotyping (including aggressiveness tests, secure high aggressiveness level, use more isolates, to ensure a higher reliability of experimental data both for resistance tests and for genetic analysis. The visual evaluation is highly important, without correct data, the FDK and DON cannot be properly evaluated. However, in later developmental stages, other genetic factor can influence disease and toxin development, therefore we cannot consider the resistance components as fully independent traits. We speak about a total resistance that contains all possible resistance components (types) and the corresponding inoculation method is spraying inoculation. As low DON should be secured, we need, besides low visual scores, low FDK and low DON values. As they can be differently regulated, care should be given to test them. As QTLs have different functions, only QTLs are suggested which can reduce FDK and DON contamination in addition to visual symptoms. The selection of the crossing partners is therefore highly important work. In QTL analyses, the heterogeneity of the population for flowering time, plant height is highly significant, it is suggested to reduce the variance, like 1 week difference between the most early and late genotype and in plant height 20–30 cm larger difference is not allowed. For this reason, it is reasonable to use parents that are closer in agronomic traits and differ mostly in FHB resistance. The highly susceptible cultivars should be withdrawn from commercial production. The newly released cultivars should have low risk to secure food safety standards and easy and profitable growing. Fungicides can protect them easier and they resist more to agrotechnology mistakes and high inoculum concentration in the debris. 

## Figures and Tables

**Table 1:** Isolate specific correlations between traits of the second resistance tests with eight isolates of *F. graminearum* and *F. culmorum*, 1994–1996. No. of genotypes = 25 [34].

Isolate	Correlations Between		
	DI/FDK	DI/DON	FDK/DON
Fc12375	0.6420	0.8756	0.6963
Fc12551	0.7449	0.8315	0.7316
Fc223	0.8037	0.8695	0.7826
Fc89.4	0.8435	0.7615	0.7854
Fg12377	0.7997	0.7949	0.8942
Fg207	0.7142	0.7545	0.8101
Fg40	0.7458	0.8399	0.7256
Fg44	0.8571	0.7388	0.7483
Mean	0.8357	0.8855	0.8088
Gen. Mean	0.7689	0.8083	0.77176
All are significant at *p* = 0.001			
Fg: *F. graminearum*, Fc: *F. culmorum*			

**Table 2:** ANOVA of the FDK data of the CM82036/Remus population.

Source of Variance	SS	df	MS	F	F AxBxC	LSD 5%
Genotype A	516,296	95	5434.7	54.02 ***	27.21 ***	3.27
Isolate B	987,904	8	123,488	1227.51 ***	618.36 ***	
Year C	73,138	1	73,138	727.02 ***	366.24 ***	
Location D	320,301	1	320,301	3183.90 ***	1603.91 ***	
AxB	257,093	760	338.3	3.36 ***	1.69 ***	
AxC	74,391	95	783.1	7.78 ***	3.92 ***	
AxD	125,158	95	1317.4	13.09 ***	6.59 ***	
BxC	257,743	8	32,217.9	320.25 ***	161.33 ***	
BxD	102,493	8	12,811.7	127.35 ***	64.15 ***	
CxD	27,953	1	27,952.7	277.85 ***	139.97 ***	
AxBxC	133,930	760	176.2	1.75 ***	0.88 ns	
AxBxD	140,742	760	185.2	1.84 ***	0.92 ns	
AxCxD	75,562	95	795.4	7.90 ***	3.98 ***	
BxCxD	101,247	8	12,655.8	125.80 ***	63.37 ***	
AxBxCxD	151,776	760	199.7	1.98 ***		
Within	347,666	3456	100.6			
Total	3,693,392	6911				

*** *p* = 0.001, ns = non significant. Red font color: interactions with Genotype A [53].

**Table 3:** Correlations between FHB traits disease index, FDK, and DON in advanced breeding lines and cultivars, Szeged, 2011–2015 [75].

Between	2011	2012	2013	2014	2015	Mean
n	129	89	111	151	94	574
FHB/FDK	0.628 ***	0.578 ***	0.467 ***	0.802 ***	0.654 ***	0.626
FHB/DON	0.566 ***	0.559 ***	0.341 ***	0.441 ***	0.341 ***	0.450
FDK/DON	0.747 ***	0.853 ***	0.630 ***	0.731 ***	0.391 ***	0.670

*** *p* = 0.001.

**Table 4:** FHB resistance test in wheat, AUDPC and FDK values in the Frontana/Remus mapping populations according to the inoculation dates, 2002 and 2004 [71].

Year	2002			2004	
Inoculation	DI AUDPC	FDK %	Inoculation	DI AUDPC	FDK %
May	Mean	Mean	May/June	Mean	Mean
10	368.44	34.91	19	151.14	37.42
13	248.83	31.39	21	172.62	40.27
15	303.21	25.80	24	169.25	31.39
21	315.18	19.38	26	440.06	43.68
-	-	-	7	831.74	35.32

**Table 5:** Mapping of FHB QTLs in the Mini Mano/Frontana population (168 DH lines including parents) for DI, FDK, and DON, means for seven experiments 2008 and 2009 [25].

Marker Map Interval	Chromosome	DI		FDK		DON		Active for
		LOD	VE	LOD	VE	LOD	VE	
wPt-800509 - wPt-2780 *	4A **	**5.73**	*14.6*	1.71	*4.4*	No ***	no	**DI**
wPt-5334 - wPt-4243	4B	**3.60**	*9.1*	1.31	*3.4*	no	no	**DI**
Xgwm205 - Xgwm156	5A	**4.73**	*12.2*	**4.71**	*12.7*	no	no	**DI/FDK**
wPt-734078 - wPt-731843	1A	**3.08**	*8.0*	**2.10**	*5.6*	no	no	**DI/FDK**
wPt-7204 - wPt-744786	6A	**3.97**	*10.5*	**5.79**	*14.2*	no	no	**DI/FDK**
**wPt-6039 - Xgwm88**	**6B**	**8.14**	***20.5***	**5.31**	*13.3*	no	no	**DI/FDK**
wPt-9925 - wPt-5922	7B	**3.52**	*10.8*	**5.67**	*13.9*	no	no	**DI/FDK**
Xgwm44 - wPt-744219	NA1	**3.33**	*8.5*	**2.16**	*5.6*	no	no	**DI/FDK**
wPt-666593 - wPt-664682	NA2	**2.21**	*7.6*	**3.18**	*11.1*	no	no	**DI/FDK**
wPt-3812 - wPt-732411	2D	0.77	*2.0*	**4.73**	*11.8*	**2.49**	*6.4*	**FDK/DON**
wPt-0934 - wPt-743601	7D	0.65	*1.7*	**3.12**	*7.9*	**3.41**	*8.7*	**FDK/DON**
wPt-5347 - wPt-2315	1B	**5.06**	*17.0*	**5.02**	*13.0*	**2.78**	*7.1*	**DI/FDK/DON**
**wPt-732882 - wPt-667765**	**2D**	**6.61**	***23.0***	**6.34**	*20.6*	**4.12**	*10.3*	**DI/FDK/DON**
Xgwm533 - wPt-3921	3B	**3.97**	*10.0*	**2.54**	*6.6*	**4.01**	*10.1*	**DI/FDK/DON**
wPt-741134 - wPt-5896	5B	**2.54**	*8.7*	**5.34**	*15.0*	**4.53**	*15.2*	**DI/FDK/DON**

* Bold text: The most effective QTL in the given group. ** Bold: LOD significant at *p* = 0.001. *** no: no response at all.

**Table 6:** Possible QTL functions with activity to different FHB traits in wheat [86].

Inoculation		DI	FDK	DON
Spraying	DI	X		
	FDK		X	
	DON			X
	DI/FDK	X	X	
	DI/DON	X		X
	**FDK/DON**		**X**	**X**
Inoculation	**DI/FDK/DON**	**X**	**X**	**X**
Point/one floret	DI	X		
	FDK		X	
	DON			X
	DI/FDK	X	X	
	DI/DON	X		X
	**FDK/DON**		**X**	**X**
	**DI/FDK/DON**	**X**	**X**	**X**

X: positive response of the given trait, Bold: the most useful combinations.

## Supplementary Materials

The following are available online at https://www.mdpi.com/2223-7747/9/12/1702/s1, Figure S1. Disease index data (%) in the methodical test for inoculation methods across years and methods according to epidemic severities, 2009–2012. Figure S2. FDK data (%) in the methodical test for inoculation methods across years and methods according to epidemic severities, 2009–2012.

## Abbreviations

**AUDPC**	**Area Under Disease Progress Curve**
cvs	cultivars
DI	disease index
Fav	*F. avenaceum*
Fc	*F. culmorum*
FDK	Fusarium damaged or scabby kernels
Fg	*F. graminearum*
FHB	Fusarium head blight
Fspor	*F. sporotrichioides*
LOD	limit of odds in QTL analysis
LSD	limit of significant difference
MAS	marker aided selection
Mniv	*Microdochium nivale, Gerlachia nivalis*
NIV	nivalenol
PE	polyethylene
QTL	Quantitative trait locus.
