# Supplementary figures and images for: Updating the Breeding Philosophy of Wheat to Fusarium Head Blight (FHB): Resistance Components, QTL Identification, and Phenotyping—A Review
Source: Plants (Basel). 2020 Dec 3;9(12):1702. doi: 10.3390/plants9121702 (PMC7761804; doi:10.3390/plants9121702)

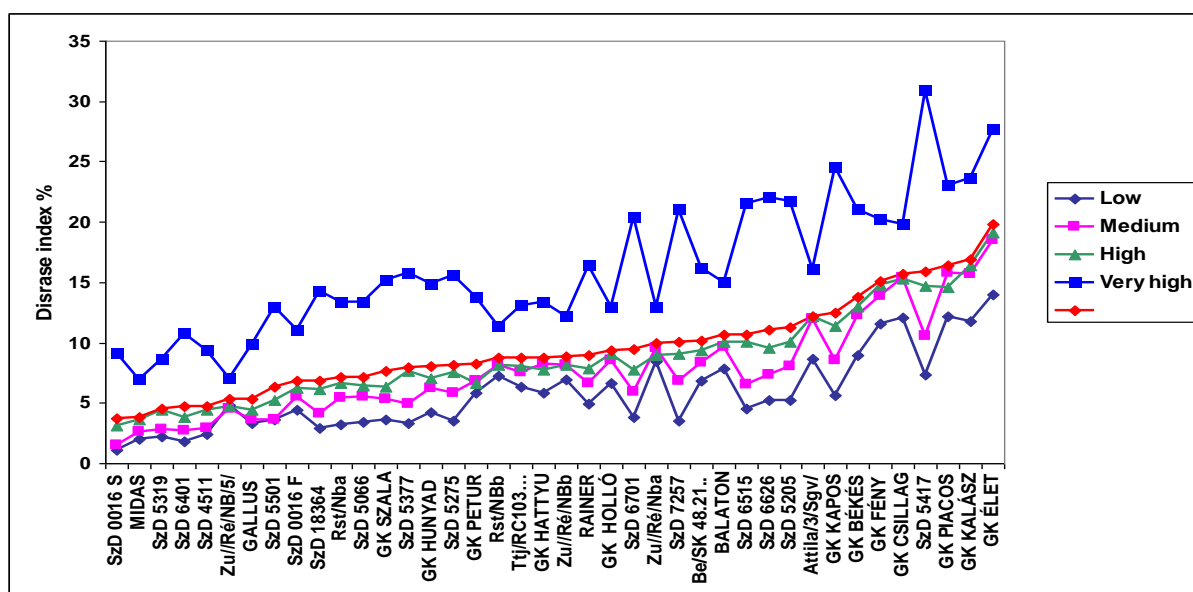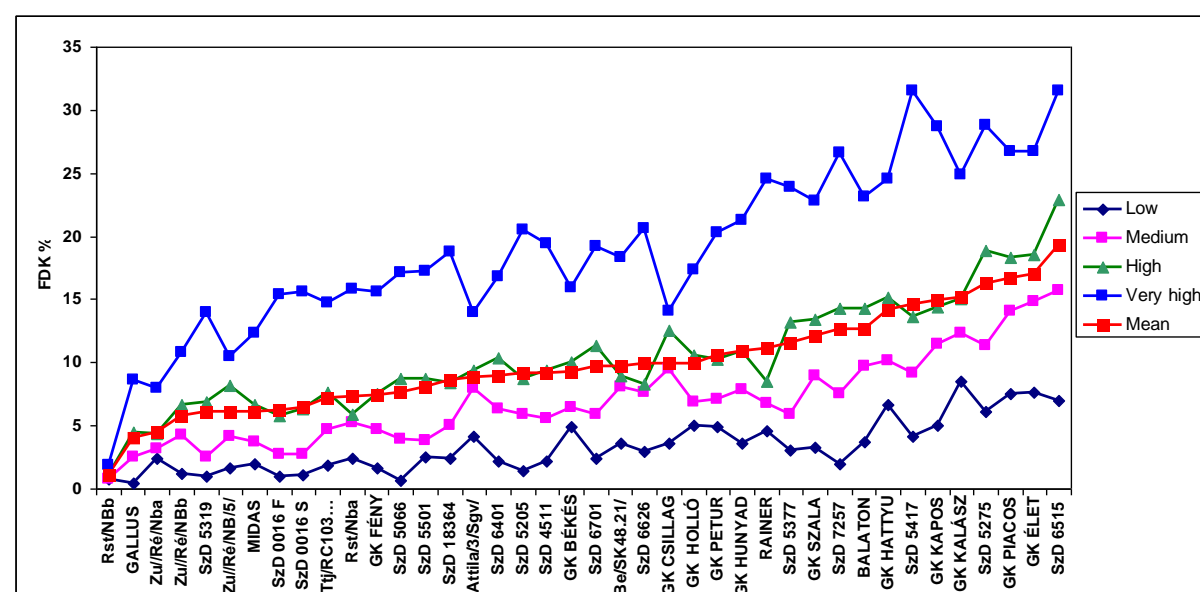

Supplement: Supplementary file 1 [file plants-09-01702-s001.pdf]
